# Complete Genome Viral Phylogenies Suggests the Concerted Evolution of Regulatory Cores and Accessory Satellites

**DOI:** 10.1371/journal.pone.0003500

**Published:** 2008-10-22

**Authors:** Paolo Marinho de Andrade Zanotto, David C. Krakauer

**Affiliations:** 1 Instituto de Ciências Biomédicas - ICB II, University of São Paulo, São Paulo, Brazil; 2 Santa Fe Institute, Santa Fe, New Mexico, United States of America; Brigham Young University, United States of America

## Abstract

We consider the concerted evolution of viral genomes in four families of DNA viruses. Given the high rate of horizontal gene transfer among viruses and their hosts, it is an open question as to how representative particular genes are of the evolutionary history of the complete genome. To address the concerted evolution of viral genes, we compared genomic evolution across four distinct, extant viral families. For all four viral families we constructed DNA-dependent DNA polymerase-based (DdDp) phylogenies and in addition, whole genome sequence, as quantitative descriptions of inter-genome relationships. We found that the history of the polymerase gene was highly predictive of the history of the genome as a whole, which we explain in terms of repeated, co-divergence events of the core DdDp gene accompanied by a number of satellite, accessory genetic loci. We also found that the rate of gene gain in baculovirus and poxviruses proceeds significantly more quickly than the rate of gene loss and that there is convergent acquisition of satellite functions promoting contextual adaptation when distinct viral families infect related hosts. The congruence of the genome and polymerase trees suggests that a large set of viral genes, including polymerase, derive from a phylogenetically conserved core of genes of host origin, secondarily reinforced by gene acquisition from common hosts or co-infecting viruses within the host. A single viral genome can be thought of as a mutualistic network, with the core genes acting as an effective host and the satellite genes as effective symbionts. Larger virus genomes show a greater departure from linkage equilibrium between core and satellites functions.

## Introduction

Evidence from complete viral genomes suggests that viruses differ from many other evolving lineages in terms of the quantity of genetic material they appropriate from other organisms as opposed to material they evolve themselves through genetic duplication and expansion events [1], [2], [3], [4]. Extensive gene transfer has lead to two alternative sets of hypotheses for the origins of viruses [5]. One set consists of escaped gene or escaped transcript hypotheses, which hypothesize that viruses are of cellular origin, and that they emerge through a form of retrograde evolution or “devolution”. The best known of these is the Green-Laidlaw hypothesis [6], [7] that views viruses as degenerate organisms. A variant is the Galatea hypothesis [8] that envisages a bacterial nucleoprotein as the viral precursor. The second set of hypotheses treat viruses as relics of primitive forms of life, typically prebiotic, nucleic acid replicators from the RNA world that became co-adapted to primitive, and subsequently, more recent forms of cellular life. It has also been proposed that DNA viruses could have infected RNA cells promoting the transition from the RNA to the DNA world [9], eventually leading to the emergence of eukaryotic cellular organization from prokaryotic cells (*i.e.*, eukaryogenesis) [10]. These theories differ with respect to the antiquity of viruses, their mechanism of origination, and their possible role in shaping cellular diversity. A characteristic of all virus origin theories is that virus genomes are thought of as considerably more fluid than those of eukaryotes and even prokaryotes, with their genetic material frequently swapped in and out during the course of evolution. In this paper we seek to show that the history of a considerable fraction of DNA virus genes can be predicted by the history of their DNA dependent DNA polymerase (DdDp), which can be traced back to host genomes. We suggest that these findings add support to an interpretation of virus evolution based on extensive sampling of host genomes by itinerant viruses. In particular, the DNA evidence suggests that viruses emerge from a core reaction network centered about the DdDp enzyme, followed by a concerted evolution of members of this network responding to a set of variable, cellular environments.

## Results

### Viral assemblages

**Figure 1:**
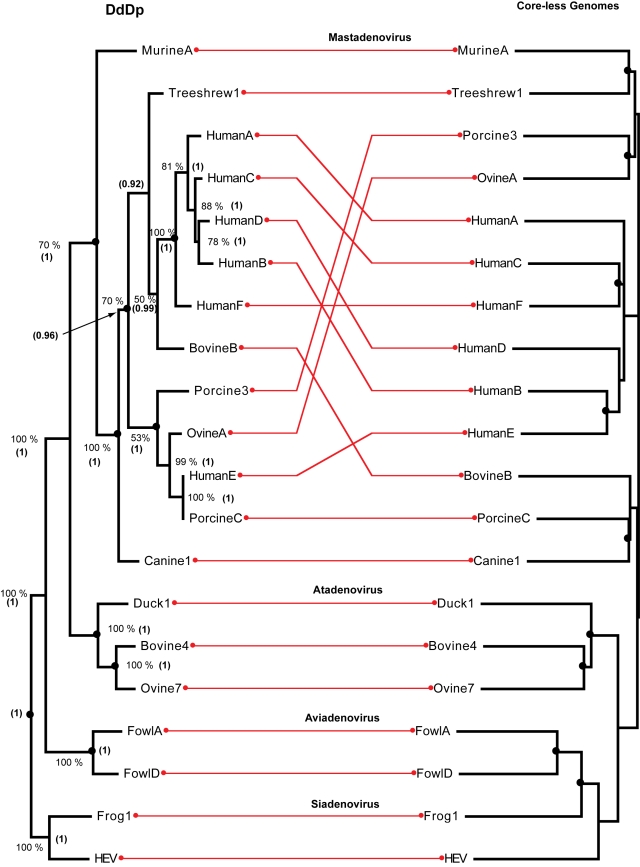
Assemblage of an adenovirus DNA-dependent-DNA polymerases (DdDp or cores) maximum likelihood tree (inferred with *phyml*) rooted at the Siadenovirus (left) and a phenogram clustered with the neighbor-joining method implemented in the Weighbor program for adenovirus “core-less” genomes, *i.e.*, without the DdDp (including satellite functions) rooted at the node connecting the Atadenovirus to the Siadenovirus (right). Nodes encircled by black dots indicate codivergence events. Values near the nodes of the DdDp indicate the number of times that each tree component was observed during 500 non-parametric bootstrap maximum likelihood iterations with *phyml*, value between parenthesis are the posterior Bayesian probability of the node estimated with MrBayes. Nodes encircled by black dots indicate codivergence events estimated with the TreeMap program.

Phylogenetic trees for the DdDp and phenograms, which are trees obtained from a complete core-less genome sequence (genomes without DdDP), were obtained through a comparison of 20 complete adenovirus genomes shown in Table 1. All principal groups of adenovirus (Mastadenovirus, Atadenovirus, Aviadenovirus and Siadenovirus) were recovered in all trees used for the assemblage (Figure 1). Table 2 indicates the observed number of codivergence events, events in which DdDp phylogenies and core-less phylogenies share an equivalent branching event. The codivergence values were optimized by means of a heuristic search using the TreeMap application that simultaneously estimates the required duplication, exchange and sorting events (reconstructions not shown) that maximize the number of codivergence events. The probability of the observed number of codivergence events given a suitable null expectation after 10 thousand randomizations of trees in each assemblage, ranged from 0.010 to 0.047 (Table 2). This suggests that the DdDp phylogeny is a strong predictor of the history for a large set of phylogenetically informative satellite genes encoded in the viral genome.

**Table 1:** Sources, names, labels and sizes for complete genomes from selected representatives from four DNA virus families.

Accession Number (NCBI)	Virus Family and Name	Taxon label	Genome size in bp
	**Adenoviridae (20)**		Mean = 33762
NC_000899	Fowl adenovirus D	FowlD	45063
NC_000942	Murine adenovirus A	MurineA	30944
NC_001405	Human adenovirus C	HumanC	35937
NC_001454	Human adenovirus F	HumanF	34214
NC_001460	Human adenovirus A	HumanA	34125
NC_001720	Fowl adenovirus A	FowlA	43804
NC_001734	Canine adenovirus 1	Canine1	30536
NC_001813	Duck adenovirus 1	Duck1	33213
NC_001876	Bovine adenovirus B	BovineB	34446
NC_001958	Hemorrhagic enteritis virus	HEV	26263
NC_002067	Human adenovirus D	HumanD	35100
NC_002501	Frog adenovirus 1	Frog1	26163
NC_002513	Ovine adenovirus A	OvineA	33034
NC_002685	Bovine adenovirus 4	Bovine4	31301
NC_002702	Porcine adenovirus C	PorcineC	32621
NC_003266	Human adenovirus E	HumanE	36521
NC_004001	Human adenovirus B	HumanB	34794
NC_004037	Ovine adenovirus 7	Ovine7	29574
NC_004453	Tree shrew adenovirus 1	Treeshrew1	33501
NC_005869	Porcine adenovirus 3	Porcine3	34094
	**Baculoviridae (25)**		Mean = 126818
AY327402	Choristoneura fumiferana defective (nucleopolyhedrovirus) NPV	CfDEFNPV	131158
AY430810	Neodiprion sertifer NPV	NsSNPV	86462
NC_001623	Autographa californica NPV	AcMNPV	133894
NC_001875	Orgyia pseudotsugata NPV	OpMNPV	131995
NC_001962	Bombyx mori NPV	BmMNPV	128413
NC_001973	Lymantria dispar NPV	LdMNPV	161046
NC_002169	Spodoptera exigua NPV	SeMNPV	135611
NC_002331	Xestia c-nigrum granulovirus	XnGV	178733
NC_002593	Plutella xylostella granulovirus	PxGV	100999
NC_002654	Heliocoverpa armigera NPV G4	HaSNPV	131403
NC_002816	Cydia pomonella granulovirus	CpGV	123500
NC_003083	Epiphyas postvittana NPV	EppoMNPV	118584
NC_003084	Culex nigripalpus baculovirus	CuniNPV	108252
NC_003094	Helicoverpa armigera NPV	HaNPV	130760
NC_003102	Spodoptera litura NPV	SpltNPV	139342
NC_003349	Helicoverpa zea SNPV	HzNPV	130869
	**Baculoviridae (cont.)**		
NC_003529	Mamestra configurata NPV A	MacoMNPVA	155060
NC_004062	Phthorimaea operculella granulovirus	PoGV	119217
NC_004117	Mamestra configurata NPV B	MacoNPVB	158482
NC_004323	Rachiplusia ou multiple NPV	RoNPV	131526
NC_004690	Adoxophyes honmai NPV	AhNPV	113220
NC_004778	Choristoneura fumiferana NPV	CfMNPV	129609
NC_005038	Adoxophyes orana granulovirus	AdorGV	99657
NC_005068	Cryptophlebia leucotreta granulovirus	CrleGV	110907
NC_005906	Neodiprion lecontei NPV.	NlNPV	81756
	**Nudiviridae (1)**		
NC_004156	Heliothis zea virus 1	Hz-1	228089
	**Herpesviridae (31)**		Mean = 156236
NC_001493	Ictalurid putative-herpesvirus 1	IcHV-1	134226
NC_000898	Human herpesvirus 6B	Human6B	162114
NC_001345	Human herpesvirus 4	Human4	172281
NC_001347	Human herpesvirus 5	Human5	230287
NC_001348	Human herpesvirus 3	Human3	124884
NC_001350	Saimiriine herpesvirus 2	Saimiri2	112930
NC_001650	Equine herpesvirus 2	Equine2	184427
NC_001664	Human herpesvirus 6	Human6	159321
NC_001716	Human herpesvirus 7	Human7	144861
NC_001798	Human herpesvirus 2	Human2	154746
NC_001806	Human herpesvirus 1	Human1	152261
NC_001826	Murid herpesvirus 4	Murid4	119450
NC_001844	Equine herpesvirus 4	Equine4	145597
NC_001847	Bovine herpesvirus 1	Bovine1	135301
NC_001987	Ateline herpesvirus 3	Ateline3	108409
NC_002229	Gallid herpesvirus 2	Gallid2	138675
NC_002512	Rat cytomegalovirus	RatCMV	230138
NC_002531	Alcelaphine herpesvirus 1	Alcelap1	130608
NC_002577	Gallid herpesvirus 3	Gallid3	164270
NC_002641	Meleagrid herpesvirus 1	Meleagrid	159160
NC_002665	Bovine herpesvirus 4	Bovine4	108873
NC_002686	Cercopithecine herpesvirus 7	Cercopit7	124138
NC_002794	Tupaia herpesvirus	TupaiaHV	195859
NC_003401	Macaca mulatta rhadinovirus	MmrhadV	133719
NC_003409	Human herpesvirus 8	Human8	137508
NC_003521	Chimpanzee cytomegalovirus	ChimpCMV	241087
NC_004065	Mouse cytomegalovirus 1	MouseCMV1	230278
NC_004367	Callitrichine herpesvirus 3	Callitric3	149696
NC_004812	Cercopithecine herpesvirus 1	Cercopit1	156789
NC_005261	Bovine herpesvirus 5	Bovine5	138390
NC_005264	Psittacid herpesvirus 1	Psittacid1	163025
	**Poxviridae (22)**		Mean = 190509
NC_001132	Myxoma virus	Myxo	161773
NC_001266	Rabbit fibroma virus	Rabbitfib	159857
NC_001559	Vaccinia virus	Vaccinia	191737
NC_001611	Variola virus	Variola	185578
NC_001731	Molluscum contagiosum virus	Molluscum	190289
NC_001993	Melanoplus sanguinipes entomopoxvirus	MsEPV	236120
NC_002188	Fowlpox virus	Fowlpox	288539
NC_002520	Amsacta moorei entomopoxvirus	AMEV	232392
NC_002642	Yaba-like disease virus	YabaMTV	134721
NC_003027	Lumpy skin disease virus	LSDV	150773
NC_003310	Monkeypox virus	Monkeypox	196858
NC_003389	Swinepox virus	Swinepox	146454
NC_003391	Camelpox virus	Camelpox	205719
NC_003663	Cowpox virus	Cowpox	224499
NC_004002	Sheeppox virus	Sheeppox	149955
NC_004003	Goatpox virus	Goatpox	149599
NC_004105	Ectromelia virus	Ectromelia	209771
NC_005179	Yaba monkey tumor virus	Yabalike	144575
NC_005309	Canarypox virus	Canarypox	359853
NC_005336	Orf virus	Orf	139962
NC_005337	Bovine papular stomatitis virus	BPSV	134431
NC_005858	Rabbitpox virus	Rabbitpox	197731

**Table 2:** Codivergence events of DdDp (core) and “core-less” genomes (satellite) assemblages for four DNA viral families.

Tanglegrams§	Codivergence Events (Cd)	p¶	Duplication events	Exchange events	Sorting events	Total non-codivergent Events (nCd)	Cd/nCd
**Adenovirus**							
**Core & Satellite**							0.147
ML & Median	9	0.0457	10	0	54	64	
ML & Mean	10	0.0105	9	0	59	68	
Pars & Median	10	0.0112	8	1	49	58	
Pars & Mean	9	0.0478	10	0	58	68	
Total events	38					258	
**Baculovirus**							
**Core & Satellite**							0.374
ML & Median	18	<0.0012	6	1	22	29	
ML & Mean	17	<0.0008	7	1	41	49	
Pars & Median	14	0.0011	11	0	49	60	
Pars & Mean	16	<0.0001	8	1	27	36	
Total events	65					174	
**Herpesvirus**							
**Core & Satellite**							0.608
ML & Median	22	<0.0001	7	1	30	38	
ML & Mean	23	<0.0001	6	1	29	36	
Pars & Median	22	<0.0007	7	1	29	37	
Pars & Mean	23	<0.0008	6	1	30	37	
Total events	90					148	
**Poxvirus**							
**Core & Satellite**							1.030
ML & Median	17	<0.0001	3	1	12	16	
ML & Mean	17	<0.0001	3	1	12	16	
Pars & Median	17	<0.0001	3	1	13	17	
Pars & Mean	17	<0.0001	3	1	13	17	
Total events	68					66	

¶ The reconstructions of codivergence events were obtained by minimizing the numbers of duplication, exchange and sorting events, inferred with the TreeMap program v 1.0, and evaluated against 10 thousand random assemblages to obtain a number of codivergent events expected by chance, which was expressed as probability of the observed the number codivergent events shown in the ***p*** column.

§ Assemblages (tanglegrams) for each virus family included trees for the core (DdDp) reconstructed with maximum likelihood (ML) and parsimony (MP) and phenomes for satellite functions (core-less genomes) using the Median and Mean distances. Cd/nCd (last column) shows the ratio of codivergence (Cd) to the sum of non-codivergence (nCd) events for each viral family.

**Figure 2:**
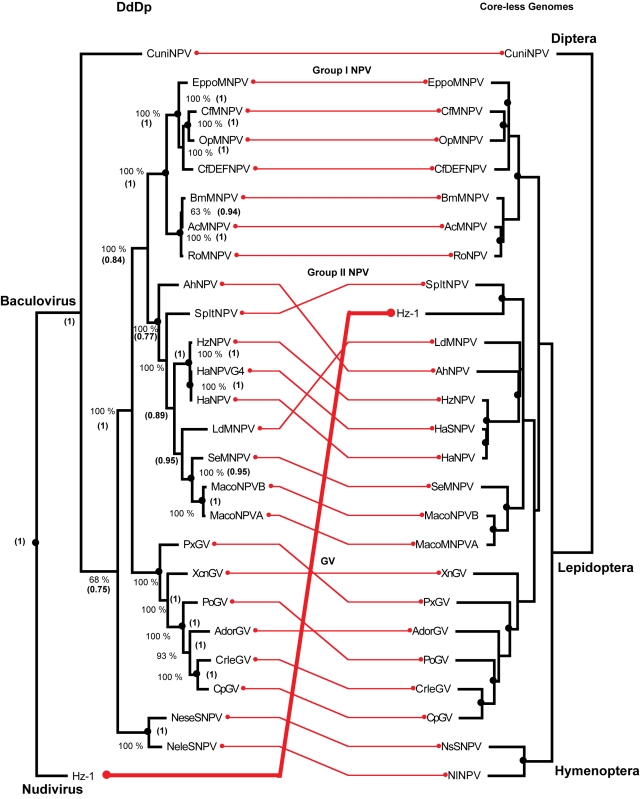
Assemblage of a baculovirus DNA-dependent-DNA polymerase (DdDp or cores) maximum likelihood tree rooted at the *Heliothis zea* nudivirus Hz-1 (left) and a phenogram clustered with the neighbor-joining method implemented in the Weighbor program for baculovirus “core-less” genomes, *i.e.*, without the DdDp (including satellite functions) rooted at the most distantly-related baculovirus the CuniNPV (right). Nodes encircled by black dots indicate co-divergence events. Values near the nodes of the DdDp indicate the number of times that each tree component was observed during 500 non-parametric bootstrap maximum likelihood iterations with *phyml*, value between parenthesis are the posterior Bayesian probability of the node estimated with MrBayes. Nodes encircled by black dots indicate codivergence events estimated with the TreeMap program.

For the 26 genomes in the baculoviruses dataset, the trees shown in Fig. 2 recover the principal associations within this clade (*i.e.*, Group I and II of the NPV and GVs) based on the polyhedrin gene [11], genome content [12] and concatamers of several orthologues [12], [13], and reaffirm the proposed position of the root of the tree [13], [14], [15]. An interesting anomaly is *Heliothis zea* virus (Hz-1), from the Nudiviridae family, that infects *Spodoptera* and has a DdDp that is highly divergent from those of Group II NPV and the baculoviruses in general [13], [14], but nevertheless clusters in the “core-less” phylogeny, shown at the right in Figure 2, as a sister taxa of the SfltNPV infecting *Spodoptera littura*. This suggests that viruses from distinct families can converge to infect closely related hosts. But by and large, the high congruence observed between both DdDp and the tree derived from core-less genomes, suggests that the history of the complete genomes recapitulates that of DdDp during adaptive radiation in insects. The probabilities for the observed codivergence events in any assemblage were less than 0.0012 (Table 2).

**Figure 3:**
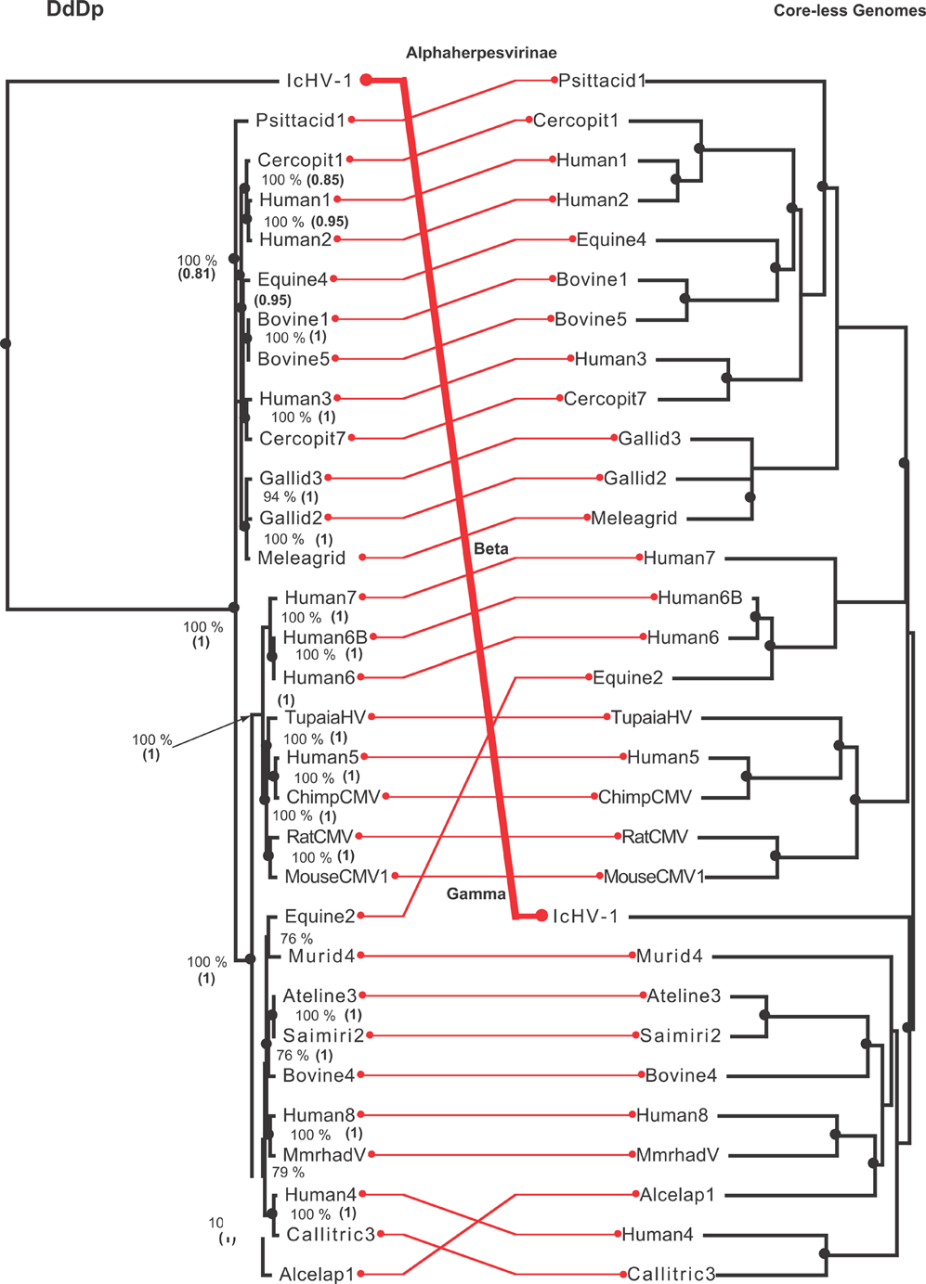
Assemblage of a herpesvirus DNA-dependent-DNA polymerase (DdDp or cores) maximum likelihood tree rooted with the Ictalurid virus (IcHV-1) infecting the Channel catfish (right) and a phenogram clustered with the neighbor-joining method implemented in the Weighbor program for herpesvirus “core-less” genomes, *i.e.*, without the DdDp (including satellite functions) rooted at the Alpha-herpesvirus (Alphaherpesvirinae) that infect birds and mammals (right). In spite of the IcHV-1 being distantly related to the herpesvirus and of questionable membership in the Herpesviridae family, it shares several satellite functions with the Beta and Gammaherpesvirinae infecting tetrapods. Nodes encircled by black dots indicate co-divergence events. Values near the nodes of the DdDp indicate the number of times that each tree component was observed during 500 non-parametric bootstrap maximum likelihood iterations with *phyml*, value between parenthesis are the posterior Bayesian probability of the node estimated with MrBayes. Nodes encircled by black dots indicate codivergence events estimated with the TreeMap program.

For the 31 herpesviruses, the DdDp trees showed a good agreement with those obtained from complete genomes. The three subfamilies *Alphaherpesvirinae*, *Betaherpesvirinae* and *Gammaherpesvirinae* were recovered for all trees using all methods, albeit with varying degree of support (data not shown). The agreement between DdDp and complete genomes corresponded to the results of a 46-taxon composite topology based on multiple genes [16]. The separation of the *Gammaherpesvirinae* into two subgroups, *Betaherpesvirinae* into two subgroups, and *Alphaherpesvirinae* into three subgroups agreed with both the 46-taxon composite topology, and a study on the gene content phylogeny of the herpes viruses using clusters of orthologous groups (COGs) of genes [1]. The ictalurid IcHV-1 infecting the Channel catfish has similar gross morphology, limited sequence conservation, and almost the same capsid architecture as that found in herpesvirus [17]. As expected, its DdDp did not nest within the three herpes subfamilies. Nevertheless the ATPase subunit of the DNA packaging enzyme complex (terminase) [18], [19] is likely to have promoted convergence with herpesvirus infecting tetrapods, (shown at the right in Figure 3). In general, as with the baculoviruses, the herpesvirus DdDP is a good predictor for the history of the rest of the genome and the probabilities for the observed number of codivergence events in all assemblages were less than 0.0008 (Table 2).

**Figure 4:**
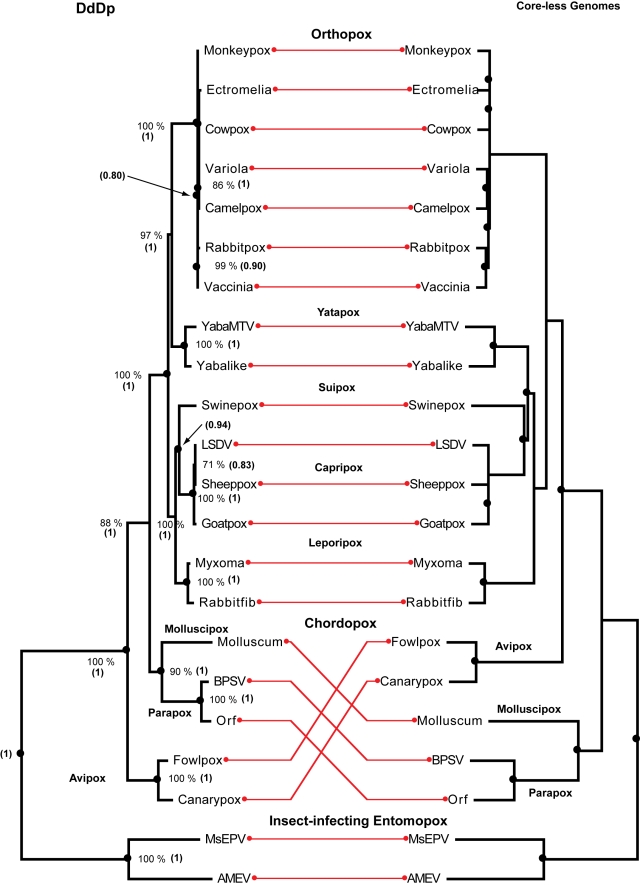
Assemblage of poxvirus trees rooted with the insect-infecting entomopoxvirus and a phenogram clustered with the neighbor-joining method implemented in the Weighbor program for poxvirus “core-less” genomes, *i.e.*, without the DdDp (including satellite functions). All major groups of posxvirus were recovered. Nodes encircled by black dots indicate co-divergence events. Values near the nodes of the DdDp indicate the number of times that each tree component was observed during 500 non-parametric bootstrap maximum likelihood iterations with *phyml*, value between parenthesis are the posterior Bayesian probability of the node estimated with MrBayes. Nodes encircled by black dots indicate codivergence events estimated with the TreeMap program.

For the 22 poxviruses (Table 1), all core-less genome trees are congruent with the DdDp tree (Figure 4). All methods recovered the separation of insect infecting (entomopox) and vertebrate-infecting (chordopox) groups. The orthopox monophyletic group within the chordopox was in good agreement with a tree constructed with 34 sets of othologous genes [2]. Moreover, the associations recovered within the orthopox are in agreement with that obtained from a set of 92 gene families [2]. Overall, the trees in Fig. 4 support earlier studies conducted with clusters of orthologues and gene content analyses [20], [2], [21]. The most notable discrepancy is the close association of the Electromelia and cowpox viruses to the variola virus as a sister group to the camelpox virus. The trees obtained for the orthopox virus support the hypothesis of infection of peri-domestic animals, allowing for independent cross-species transmission into the human species. Intra-group resolution among orthopox has poor statistical support but tends to agree with a previous study proposing that this group of viruses have had a zoonotic origin [2]. The pox viruses has the largest genome (Table 1) and the best agreement between the histories of core and satellites (Table 2), with the probability of observing the reported number of codivergences less than 0.0001 for all assemblages.

### The dynamics of gene gain and loss

**Figure 5:**
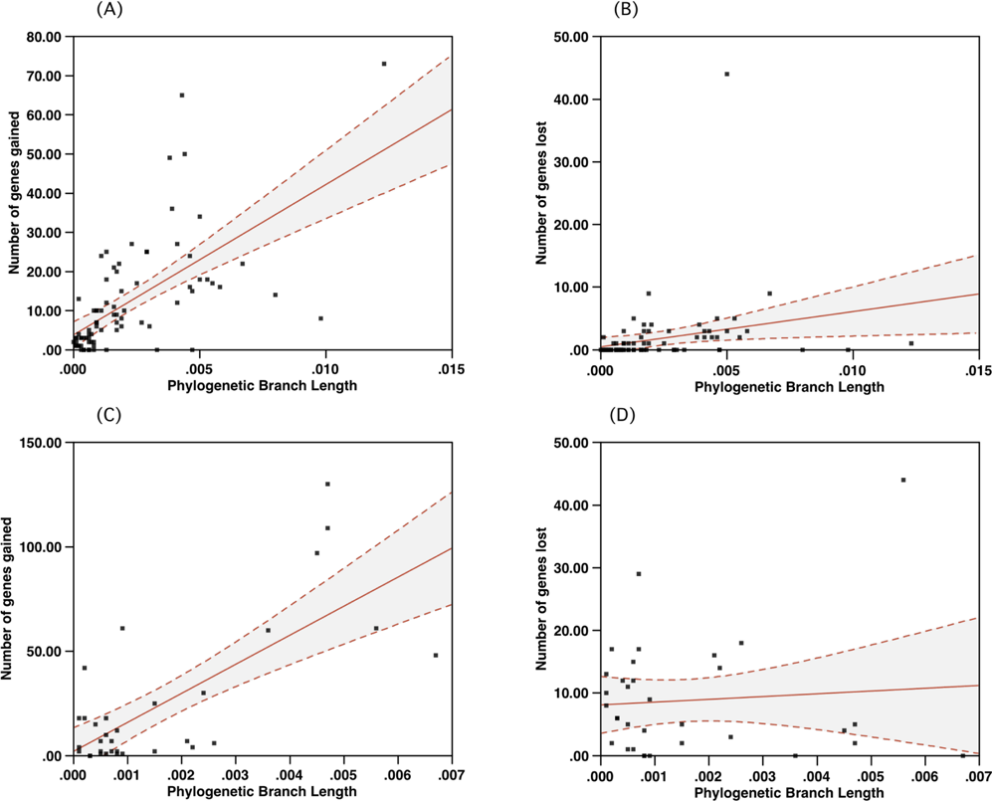
Gene accretion and loss in the baculovirus (5A and 5B) and poxvirus (5C and 5D). Solid lines are least squares linear regression to the data. Dashed lines bound 95% confidence intervals on the mean regression lines. Rates of gene gain are far higher than rates of gene loss. The number of gain and loss events for baculovirus was estimated with MacClade and for poxvirus was obtained from McLysaght *et al.*, [2]. The regression indicate a positive dependence on the number of gene gains on branch length in the tree for the complete genomes, whereas loss events were less frequent and had no significant dependence on branch length (as indicated by the low correlation coefficients on the shallow gradients). The data suggested that the process of gene gain in these 2 viral families is temporally organized, since genetic distance (branch lengths) is proportional to time. For both loss and gain the distribution of gain and loss departs from a simple exponential model predicted by the branch lengths. A similar accretion process of auxiliary gene functions in large DNA viruses has been observed for herpesvirus [1].

Our data strongly supports the notion that viral genomes are organized around a core set of genes, such as those encoding the replication machinery. We also investigated the gene accretion process by studying gene-content variation among distinct viral families. We considered the number of gene gain events along a lineage or branch of a phylogeny versus the branch length (considering only those branches that experience gene gain and loss events) for complete genome phylogenies estimated with BlastPhen for both, baculoviruses [15] and poxviruses [2] (Figures 5a and 5b). We found that the rate of gene gain in the poxviruses was around four times the rate of gene gain in the baculoviruses. The rate of gene loss in both of these groups was significantly lower than the rate of gain, and while most losses are concentrated in shorter branch lengths, the loss rate remains effectively independent of the time over which we have been able to estimate branch lengths. The ratio of gain to loss of baculoviruses was approximately 7, whereas the ratio for the poxviruses was around 30. However the variance in these estimates was very large (Figure 5) with coefficients of determination (*r^2^*) on the gain regressions of 0.40 for 835 gene-gain events in baculovirus and, 0.55 for 261 gene-gain events in poxvirus. The values were significantly larger than the range of 10^−2^ for the coefficients of determination of loss regressions, with values of 0.006 for 135 gene-loss events for baculovirus and 0.07for 230 gene-loss events in poxvirus. The results suggest that 50% of the variability observed in gain events was explained by a linear model, while there was no detectable dependency of loss events on branch lengths (*i.e.*, time). Moreover, the positive relationship of the gain event against time, as determined by Pearson's coefficient (*r*), for log-transformed gain-events versus log-transformed branch lengths was 0.73 for baculovirus and 0.49 for poxvirus, which further supports the idea that gain events are time ordered, and potentially, stereotypically sequential. The asymmetry of gain to loss indicates that for both of these groups, the genomes show a significant net expansion through time, with many new genes fixing in the populations. Interestingly, the insect-infecting entomopox viruses accreted 239 genes and lost only 5 genes along 2 lineages. On the other hand, significant genetic exchange took place along the lineages infecting higher animals (*i.e.*, Chordopox and Orthopox genus) [2].

## Discussion

### Phenomics sheds a light on DNA virus genome evolution

Extensive comparisons between a whole genome quantitative treatment, or “phenomic” approach to virus genomes, and a detailed sequence alignment approach based on a highly conserved polypeptide (*i.e.*, the DdDp), suggests that un-annotated (FASTA formatted) DNA sequence data from complete viral genomes provides a source of useful information about viral phylogenies at a significantly reduced computational cost [14], [15]. The phenomics approach has an additional benefit of minimizing errors arising from ambiguities in the order of genes. An important result was the strong agreement (*i.e.*, congruence) among the complete genome quantitative measures, or phenetic clusters (phenomics), and the DdDp maximum likelihood phylogenies for all 4 viral families studied. Moreover, this agreement had a strong historical, or homologous component, made evident by the frequent co-divergence of large stretches of the genomes, and a significant convergent component, realized through concerted gene gain events. This degree of agreement can seem surprising when we think of viruses as itinerant regulatory networks [25]. These are networks that expand their functional repertoires as they acquire new host genes along their evolutionary history (either directly from the host or indirectly from co-infecting viruses) and thereby come to increasingly resemble host genomes. We might expect therefore significant convergence among genomes that does not reflect true ancestor-descendent relationships (as in the case of the IcHV-1 to herpesviruses and Hz-1 to baculovirus). For most of the sequence data that we consider, we have observed congruence reflecting conservation at the level of the whole genome.

Given the potential for horizontal gene transfer among viruses and among viruses and host cells, in contrast to the stability of DdDp orthologues shared among viral families [13], [22] and host cells, the congruence becomes an issue in itself. It requires that we rethink the *process* of viral origination and evolution, outside of the traditional molecular systematics interpretation of patterns of adjacency on trees, which treat large-scale similarity as evidence of inheritance from con-specific ancestors. Here we shall argue that large-scale genomic similarity among viruses derives in part from homology among the repertoire of host genomes. This has two components: a core component, reflecting an early escape of the core, conserved polymerase gene from one of two host lineages, and a satellite component, reflecting the later concerted evolution of viruses in common host environments with an overlap in the pattern of genetic accretion. *What is significant is that the accretion of satellite functions remains largely congruent with the polymerase core*, *and thus tends to retain the “true” phylogenetic signature as revealed by the core*. This is in contrast to the expectation for convergence, which is typically thought to disrupt historical signatures by virtue of the diversity of the pool of potential, host environments.

In this work, we focused on DNA viruses because they use 2 types of DdDp (A and B) of known cellular origin [13], [22] and they range several orders of magnitude in genome size (from parvovirus with few thousands of base pairs to giant algae viruses with millions base pairs), which makes the process of gene accretion very interesting to investigate. On the other hand, RNA viruses (with the exception of retroviruses) possess an RdRp not shared with the cell and their genomes are limited to few tens of thousand base pairs (the largest being the coronaviruses with 30 Kbp genomes), which often can be aligned allowing conventional phylogenetic analysis to be performed without the need for “alignment-free” methods.

### Origins of polymerase and congruent genomes

The adenoviruses have a Type A DdDp, which is related to the polymerase found in the bacteriophages, which suggests a prokaryotic origin for this group. On the other hand, the large DNA viruses from the remaining three families presented in this study have Type B DdDp, with orthologues in eukaryotic cells [13], [22]. The question naturally arises whether the evolutionary history of DdDp is representative of the behavior of a large set of genes in the genome or whether it is anomalous. It is possible after all that viruses have engaged in core-exchanges, promoting a false homology for the majority of genes in the genome. The strong agreement we observed between the sequence-based approach and the whole genome, phenomics approach, supported the idea that a large set of genes in each of the virus lineages have had congruent phylogenetic histories. By clustering all 99 genomes in this study with additional representatives of other DNA families (data not shown), it was observed that Type A and Type B DdDp families separate into distinct clusters, further establishing the role of the DdDp as a major discriminatory gene among viruses. Moreover, the adenovirus had the worst agreement of core and satellite phylogenies, whereas the largest genomes (poxviruses) had the best agreement. The fit of the average genome size (Table 1) to the codivergence probability values (Table 2) for all four viral families had a significant negative dependence (*r^2^* = 0.865, linear, 0.994 exponential and, 0.971 power) which might indicate that smaller genomes experience reduced epistasis and thereby have a greater capacity to accrete novel gene functions.

### Considering genomics cores and satellites as hosts and symbionts

**Figure 6:**
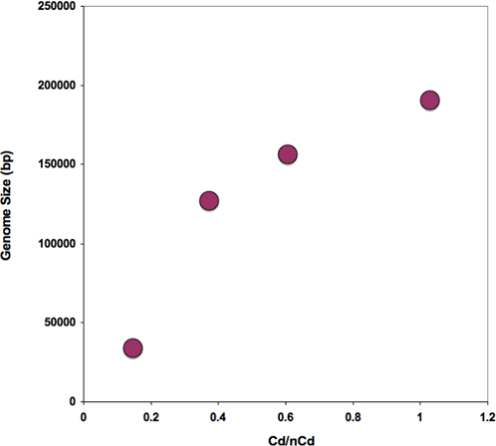
Plot showing a positive correlation (*r^2^* = 0.84) between the average genome in each viral family versus the ratio (Cd/nCd) of codivergence (Cd) over non-codivergence (nCd) events. The ratio is a measure of the linkage disequilibrium between the core polymerase gene and the remainder of the genome. Larger genomes tend to diverge in a more concerted fashion, whereas smaller genomes show greater independence in genetic segregation among constituents of their genomes. Hence smaller viral genomes behave somewhat like a mutualistic network, with the core acting as a host and the accessory satellites as symbionts.

We have examined congruence among polymerase-based trees and core-free, whole genome trees. A significant proportion of correlated branching patterns in these trees can be attributed to a shared evolutionary history–codivergence events. However, there are many, non-codivergence events (duplications, exchanges or switching, and sorting) required to maximize the incidence of co-divergences between the core and its satellites (when maximizing similarity through common descent). These could always indicate noise resulting from systematic errors in inference, but the high bootstrap values on the DdDp trees suggests otherwise. These events are perhaps better understood as a measure of gene flux in the viral GRN. *If we assume that the DdDp is the crucial organizing component of the virus genome*, *we might think of satellite genes as essentially commensal or mutualistic components in relationship to the core*. Thus a single genome, when significantly fluid, might be thought of as a mutualistic network. When considering the relationship of hosts and symbionts, co-divergence reflects a shared evolutionary history. When hosts undergo speciation with their symbionts, their reconstructed trees will tend to be congruent. However, symbionts can also gain access to new hosts by switching among them. This promotes a departure from congruence. It is also possible that when a host that is infected with multiple strains of symbionts undergoes speciation, that only a single symbiont is able to infect daughter species. Thereby only one lineage is preserved during sorting. Similarly if symbionts are duplicated and diverge within a host, and subsequently associate differentially with daughter species, this will promote departure from congruence. Incongruity between the core and the satellites could arise through all of these processes, but the identification of the effective host and effective symbiont, is a little less clear. Switching, sorting and duplication could refer equally to variation in the core and the satellites. New genes can accrete around an old core, or a core could displace another core and occupy the satellite niche of that core. These alternative accounts for incongruence can only be discriminated by examining the history of each lineage in detail. However, DdDp exchange among viruses is expected to be more common among closely related lineages, because as viruses diverge, the exchange of unrelated DdDp would require the concerted exchange of a number of cofactors. For this reason we might think of the core as the effective genomic host. Without considering the detailed histories, we can derive an approximate measure for genome flux at a coarse grain, by calculating the ratio of codivergence events (Cd) to all possible events (codivergence, sorting, duplication and switching) added together (nCd) for a viral group. We observed that the ratio Cd/nCd of codivergence (Cd) to non-codivergence (nCd) events was strongly related to the average viral genome size across the viral families (*r^2^* = 0.84) (Figure 6). Hence larger genomes tend to show stronger linkage disequilibrium between the core and its satellites. This suggests stronger dependencies, or epistasis, between the core and the satellite as the number of functions increases.

### Core and satellite functions

The hypothesis of a conserved, co-evolving set of genes derives additional support from gene content studies on DNA viruses. For example, baculovirus gene content studies show that a core set of 30 genes are shared among baculovirus, some of which, such as *helicase* ac96, 38K(ac96) maintain similar relative positions in the genomes [4]. One third (10) of these conserved genes are involved either in replication (4) or transcription (6), whereas many others are an assortment of structural proteins (9), and auxiliary (1) or unknown function (10). This indicates that most conserved functions shared among known baculovirus are related to the replicative/multiplicative core. Our whole genome trees (data not shown) agree with previous phylogenies of the baculovirus based on single genes [11] or with clusters of orthologues [4], [13]. Likewise, in the poxviruses, a core set of 34 genes involved in replication and viral assembly were found to be present in the 20 genomes studied. The orthopox genomes have an additional set of 52-shared genes [2]. In the latter study, it was proposed that poxvirus genomic evolution takes place under heterogeneous rates of gene gain and loss along distinct lineages, whereas a core set of 34 genes defines the functional or metabolic identity of the virus family. In the case of the herpes virus, the core set of genes has been studied in detail. Like baculoviruses and poxviruses, the largest core set of genes: 57% of the 14 COGs including all herpes genomes (excluding the ictalurid herpesvirus) are related to DNA metabolism [1]. An additional study on the frequency distribution of distinct functional classes of 19 herpes genomes suggests that most of the core sets of shared genes are involved in nucleotide metabolism and DNA repair, structural support (capsid and tegument) and replication [23]. In each of these case studies, it can be observed that a core set of genes and functions – typically related to replication and encapsidation – are conserved whereas auxiliary functions, possibly of contextual adaptive value, can be gained and lost in time as viruses co-evolve with their preferred hosts. However, as this core increases in size, through a translocation of satellite functions into the core network, it is expected to restrict the host species the virus is able to successfully infect. In a similar vein, transposon inactivation studies seeking to determine the minimal genome of a parasitic mycoplasma (*M. genitalium*) [59], indicate a high level of redundancy in gene functions related with replication, DNA repair and transcription, with other cellular functions largely lost. A more inclusive analysis of parasitic mycoplasma species [60] suggests that gene products available directly from the host may become neutralized when liberated from normalizing selective constraints, whereas functions under-represented in the host, tend to be preserved. This suggests that the core/satellite distinction provides a general framework for dichotomizing the genome. The core constitutes a minimal, conserved set of functions, whereas the satellites encode functions, which are strongly environmentally mediated. This dichotomy is somewhat similar to essential and non-essential genes, but stresses the conserved, phylogenetic value of the core, and the shifting and dynamical nature of the accessory genes as they move in and out of the core's orbit.

### Distinct viral families may share satellite functions

The similarities in the gene accretion process among different DNA viruses infecting insects suggested that the host genetic regulatory network (GRN) creates a common selection pressure for convergence by means of satellite functions of locally adaptive value. The baculovirus, poxvirus, iridovirus, nudivirus and Ascovirus families possess B-type DdDp adjacent to cellular B-type DdDp, distinct from those in possession of A-type DdDp [24], [13], [14]. Hence insect-infecting viruses, carrying a B-type DdDp, are likely to have accreted GRNs around regulatory cores emerging from B-type cellular hosts [25]. These 5 families in addition to infecting closely related hosts share an unusually high number of supplementary or satellite genes. For example, baculovirus share 25 genes with entomopox, 13 with the nudivirus and 14 with iridovirus (list available from the authors). The information available on these shared genes suggests that almost half are of unknown function, several are involved in apoptosis, several have tissue-specific roles, a small fraction are involved in RNA metabolism (*e.g.*, the sharing of both subunits *lef*-4 and -5 of the RdRp of the baculovirus with nudivirus), one is a putative helicase homologue, and that some are enzymes involved in cell regulation (*e.g.*, methyl-tranferases and protein kinases). None appear to have homology to capsid proteins or are directly involved in DdDp activity (data available from the authors). Thus in addition to genome similarity derived from common descent (homology) there is additional similarity that evolves as the result of an overlapping host range (analogy) which serves to reinforce, rather than diminish, the historical signal.

Some regions of the viral genomes are prone to high levels of recombination and gene shuffling. For example, highly repetitive genomic regions (*hr*) are known to be associated with loci of genome instability and divergence among baculoviruses [26]. One of the features of these unstable regions is that they tend to flank genes that have experienced duplications in several baculoviruses. These regions constitute nearly 34 distinct genes [14], and are loosely grouped as Baculovirus Repeated Open reading frames (BRO genes) [27]. BRO genes suggest a putative mechanism for satellite function accretion, whereby features such as *hr* facilitate xenologous gene exchange. Baculoviruses share at least 17 distinct BRO lineages with the ascoviruses that also infect insects. The mechanism by which genes may be acquired could entail direct exchange among distinct viruses while infecting the same host [47] or direct acquisition from the host. The first alternative is perhaps less likely, as it requires superinfection to take place at the cellular level. This is generally considered a less frequent event than independent encounters between different non-persistent viruses (such as most of the ones we addressed in our study) with phylogenetically related hosts. This remains an important empirical issue to be resolved.

### Are viral genomes examples of hypercyclic organization?

**Figure 7:**
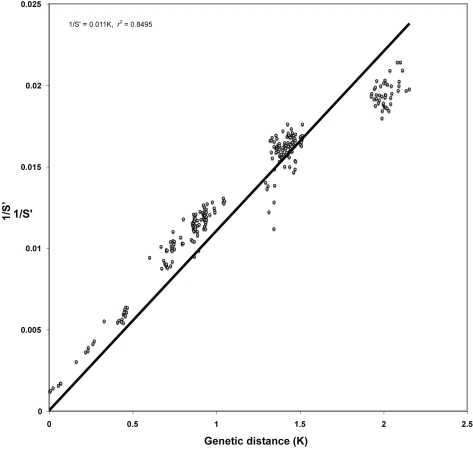
Plot of phenetic distance (1/S′) versus genetic distance (K) for baculoviruses. K was estimated from a protein concatamer of the 29 genes conserved in 24 complete baculovirus genomes (excluding the CuniNPV). K was obtained using phylogenetic ML reconstruction. 1/S′ was estimated using BlastPhen. 85% of the data was explained by the regression supporting the use of 1/S′ as a practical measure of genetic diversity. The measure is however unable to correct for superposition as K increases above 1.5. Given *k*≅φ/*S*′, φ was estimated to average 82.91 for these data.

Why should a set of genes remain so closely linked through evolutionary time? Eigen and Schuster's hypercycle [28] provides a principle of organization, by which a stable replicative, metabolic core (to include in the case of viruses those elements essential for horizontal transmission), exhibit continuity and coherence through evolutionary time. The hypercycle refers to that set of proteins constituting a closed catalytic network. By invoking the hypercycle structure of networked, genetic dependencies we go some way towards explaining the finding that the complete genomes of viruses have histories which are well represented by their replicative core, the central hub of which is polymerase. We suggest that by treating the whole genome as a quantitative variable and deriving a genomic phenotype (phenome), we are able to observe epistatic effects among genes that lead to linkage disequilibrium among members of the genomic core. This core cannot be perturbed without rendering the virus defective, and hence a large group of genes exhibit concerted evolution and, as suggested by Figure 7, it is possible that the larger the genome the greater the correlation across the genome. Nevertheless, the process of gene gain and loss at the periphery of the genomic core is likely to promote adaptive plasticity through gene exchange in locally variable, selective environments. This might be expected to weaken the historical signal from the core, whereas in our study, this process often serves to amplify the core history by virtue of overlapping host affinity.

### Phenomics and genome evolution

Virus phenomes prove to be informative much the same way that eukaryotic phenotypes can be informative, by indicating tightly coupled genetic dependencies. These dependencies can be used, when cross validated against homology, to reveal the non-overlapping evolutionary histories of complete virus genomes. In particular, the apparent independent origin of the adenoviruses from ancestral prokaryotic hosts (both sharing A-type DdDp), from that of the baculoviruses, herpesviruses, and poxviruses from eukaryotic hosts (all sharing a B-type DdDp). In general, these data are in support of the “escaped gene” hypothesis, which posits distinct virus lineages as having independent origins. We have gone further in suggesting that multiple genes might have escaped simultaneously, or as is more likely, in concert and convergently, centered about distinct polymerase catalytic cores. We investigated the gene accretion process around the catalytic cores, and demonstrated that this process shows an asymmetry in gene gain and loss in favor of gain. The gain bias can lead viral genomes to become analogous in gene content when infecting related hosts.

Congruence between two levels of genetic coarse-graining supports the hypothesis that distinct DNA virus families evolve their genomes around replicative cores derived from independent and divergent cellular origins. Crucially, the loss of genomic synteny (*i.e.*, collinearity) in evolutionary time, even when a core set of shared genes is maintained, suggests that a virus should be defined in terms of a core set of genes essential for survival that exist in a constant state of flux upon a changing background of conserved, host gene-regulatory-networks. The virus is effectively a rather diffuse organism and includes elements of the host. This dependency can promote the persistence of a phylogenetic signal in the face of significant gene transfer when the transfer process reinforces host preferences imposed through constraining core functions.

## Methods

### Complete genome sequences

The viral families and genomes used are shown in Table 1. The choice of viral families was based on the criterion that these families cover one order of magnitude in viral genome size, with adenovirus genomes of around 30 Kbp and poxvirus 200 Kbp. We used: (*i*) 20 adenovirus with a maximum length of 45,063 bp and a mean size 33,762 bp, (*ii*) 24 baculovirus together with a the *H. zea* nudivirus (Hz-1) (25 genomes total) with a maximum length of 178,733 and a mean size 156,040 bp, (*iii*) 31 herpesvirus with a maximum length of 241,087 and a mean size of 156,236 bp and, (*iv*) 22 poxvirus with a maximum length of 359,853 bp and a mean size of 190,509 bp.

### Local similarity

Our rationale was to have a measure of overall genome similarity, which avoids the need for complete genome alignments [14], [15], [49], [50]. This is because, as complete genomes diverge in time, rearrangements increase differences in gene order among taxons leading to significant alignment problems. This tendency is compounded by an increase in differences in gene content, with a net loss of shared orthologues. The loss of collinearity and orthologues through evolutionary time tends to render exhaustive alignment useful or practical only for closely related genomes. This is the case even when large amounts of information from orthologous genes are available for non-collinear, closely related genomes. Because most information at the DNA level is maintained in the coding regions of shared orthologues, it leads to little loss of information to use the translated peptides to conduct local similarity searches [55], [56], this have lead to development of several alignment-free distance and similarity metrics as well as phylogenetic reconstruction methods [49], [50], [51], [52], [53], [54]. For each combination of paired genomes, rather than attempt alignment, we used the portable implementation of **tblastx** [29] available in the NCBI Blast distribution (http://www.ncbi.nlm.nih.gov/blast/download.shtml). We installed BLAST on our computer cluster, including 60 Opteron cores running LAM/MPI 7.1 (Local Area Multicomputer / Message passing interface) (http://www.open-mpi.org/). For each pair of genomes, bit scores *S*′ [29], [30] were calculated for all local high-score pairs (HSP), that were collected to obtain a global genomic similarity either by central tendency statistics (mean, average and mode) or by comparing *S*′ distributions (see details below). Since *S*′, being a measure of similarity is obviously inversely proportional to evolutionary distance we assumed 1/*S*′ as a measure of evolutionary distance (*k*), following the approximate relation *k*≅φ/*S*′ (with an unknown proportionality factor φ. For the **tblastx** program, preliminary evaluation with limited sets of sequences indicated that both BLOSUM62, BLOSUM45 and several other transition matrices provided similar results and that collecting either 200, 500, 2000 or 40000 HSPs would not change significantly the *S*′ distributions. We also observed that using the low complexity filtering option [31] did not alter significantly our results. Other **tblastx** parameter values used were: Open Gap = 4, Gap extension penalty = 2, GapX_dropoff = 50, expect = 10 and word size = 3 (http://www.ncbi.nlm.nih.gov/blast/producttable.shtml). The **tblastx** outputs were parsed with BlastPhen (script in PERL available from the authors upon request) and the distributions of *S*′ were obtained for each pair of genomes. These distributions were subjected to a range of data analyses prior to clustering and their efficacy in generating informative genome clusters evaluated. Some statistics we calculated included moments of the distance distributions, and Kullback-Leibler or Chernoff distances among distributions. Reconstructions based on these whole genome distance metrics were then compared to phylogenetic reconstructions based on the DNA polymerase (DdDp) genes, the alignments of several orthologues or, in some cases, fully aligned complete genomes, indicated that central tendency measurements such as the mean and median, used during this work, would be sufficient to convey the main associations among genomes (data not shown).

### Clustering metrics and their properties

Most clusters obtained for the four viral families were in good agreement with pre-established phylogenies and demonstrate a strong correlation with known viral biology. The statistic *S*′ is a measure of similarity, which takes into account: (*i*) distance estimation based on amino acid transition matrices (e.g., BlOSUM, PAM, etc.) and (*ii*) a “penalty” system that considers indels (gaps caused by insertions or deletions during HSP evaluation). It is clear however that *S*′ compounds 2 types of quantities that share neither an explicit mechanism nor metric, since indels should not be treated in the same way as amino acid replacements matrices in evolutionary models of change. Nevertheless, the strong agreement among topologies built by using 1/*S*′ (reciprocal of distance or similarity) and those built from amino acid sequences of viral DNA polymerases, support the notion that 1/*S*′ is proportional to genetic distance *k*, possibly approaching *k*≅φ/*S*′, where the proportionality factor (φ) should vary according the level of congruence of the tree obtained for proteons or single genes and full genome dendograms. For example, under the circumstance that both the proteon (or gene) tree and the genome dendogram are identical with identical branch lengths along all branches, φ will be 1 and *k* will be 1/*S*′.

In the case of the baculovirus, a plot of phenetic distance (1/S′) versus genetic distance (K) had an excellent agreement (*r^2^* = 0.85) with the genome-based clusters, further supporting *k*≅φ/*S*′ (Figure 7). K was obtained using phylogenetic ML reconstruction with the Tree-Puzzle version 5.2 program [58] estimated from a 16803 amino-acids long protein concatamer for the 29 genes conserved in 24 complete baculovirus genomes. 1/S′ was estimated using BlastPhen. Most of the data fits the regression function supporting the use of 1/S′ as a measure of genetic diversity [14], [15]. Nevertheless, since 1/S′ lacks correction for superimposition when K increases above 1.5, 1/S′ can overestimate K when comparing closely related baculovirus genomes. Hence tblastx can inflate HSP from non-coding regions [15]. Having stated these reservations, given *k*≅φ/*S*′, φ averaged 82.91 for the baculovirus data. It follows than, that when comparing 1/*S*′ distribution moments such as mean, median and mode, the relationship with genetic distance is positive, because both 1/*S*′and *k* grow in time. The use of estimators of central tendency for the distance distributions (*i.e.*, low order statistics) is useful and renders a strong agreement with gene phylogenies for all datasets. Nevertheless, these low order statistics may loose information, since potentially important data provided by the distribution shape is lost.

### Inferring DdDp trees

The DdDp gene was used for phylogenetic inference to deduce the relationships among distantly related DNA viruses and among DNA viruses of the same family [22], [32], [13]. This has proved to be the best choice for an orthologue shared by all members of the viral families. Therefore, the peptide (amino acids) sequences encoded by the DNA-dependent-DNA-polymerase (DdDp) gene of each taxon were aligned with ClustalW [33]. These were used for phylogenetic reconstructions and non-parametric bootstrap using maximum likelihood (ML) method implemented in *phyml* (Guindon and Gascuel, 2003) using the WAG model plus gamma and invariant (WAG+Γ+I) as the best-fit model of the viral DdDp evolution that was determined using the Bayesian information criterion (BIC) implemented in ProtTest version 1.2.6 [34]. The robustness of each tree component was accessed by 500 non-parametric bootstrap iterations with the *phyml* program under the WAG+Γ+I model of protein evolution. Tree topology, the shape parameter (α) of the gamma (Γ) distribution of variable rates and, the proportion of invariable sites (I) were optimized during successive iterations. Moreover, we used Bayesian inference (BI) to calculate the posterior probability of each node of the DpDp trees using the parallel version of MrBayes v. 3.1.2 [35] running 20 million generations along 10 chains. Posterior probabilities of each node were calculated with the TreeAnnotator v. 4.7.7 [36] from 20 thousand trees sampled with a 10% burn-in, which was well after the sampling regime was stable around the log-likelihood (-lnL) maxima as determined by inspect the run trace behavior with TRACER v1.4 [37].

Nevertheless, since the DdDp genes within a viral family tend to maintain similar biochemical functions provided by collinear clusters of short conserved domains [22], embedded in regions that will vary dramatically among different lineages, one could argue that rate heterogeneity in time (heterotachy) could induce systematic errors during parametric ML inference [38], [for a critique see 39]. Therefore, we used a non-parametric parsimony method, as an alternative to try reducing potential bias during phylogenetic inference and as source for comparison with parametric methods such as maximum likelihood and Bayesian inference. Nevertheless, the use of parsimony under no assumption of weighted character-state change violates the assumption of a biochemical basis for preferential amino acid substitution process in time. To deal with this, accepted-mutation stepmatrices were calculated directly from the DdDp gene alignments for each viral family using the amp program (program ftp://oeb.harvard.edu/rice/amp.tar.Z.), to try establishing a realistic estimate of the minimum number of character state changes using parsimony.

### Tree reconciliation

To investigate the dependence of the history of the GRN functions [25] encoded in a viral genome to the replicative core, we reconciled the DdDp phylogeny with that of the rest of the viral genome (*i.e.*, by making a “core-less” genome excluding the DdDp from the complete genomic sequence) and evaluated how the DdDp tree predicts the phylogeny for the complete collection of satellites for each genome. Trees from complete genomes were obtained by clustering 1/S′ distance matrices using the Weighted Neighbor Joining method (WB) [40] and compared with clusters obtained with the neighbor joining (NJ), UPGMA, Fitsch (F) and Kitsch (K) methods from the PHYLIP package [41]. Trees for core (DdDp) and core-less genomes containing satellite functions were reconciled using the TreeMap V1.0 program that maximizes the number of codivergences, which are cospeciation events indicated by congruent nodes present in both DdDp and core-less genomes trees (*i.e.*, joint cladogenesis) using parsimony optimality criteria [42], [48], [49]. Tree reconciliation envisages mapping the core-less genome tree to that of the DdDp tree to estimate the historical associations between these two phylogenies. If both the DdDp and “core-less” genome trees are identical (*i.e.*, had perfectly congruent trees), the DdDp tree is a sufficient predictor for the genome. However, since both phylogenies are not necessarily identical, the reconciliation process makes uses additional events to maximize the number of co-divergences [48], [49]. These events include: (*i*) duplication, a generic term to cover a situation where tree reconciliation requires that a core-less-genome lineage speciates independently from the DdDp lineage or that we failed to find it; (*ii*) exchange or switching event which are synonyms that cover the situation where tree reconciliation requires that satellite functions associate with a number of distinct DdDp lineages and; (*iii*) sorting, where tree reconciliation requires that a core-less branch fails to follow a DdDp branching event. Moreover, it was necessary to evaluate whether the observed congruence structure and associated deviations could be explained by chance. We did this by investigating the departure from a null model capturing the number of codivergent events in the assemblage of reconciled trees for both DdDp and core-less genomes. The number of codivergent nodes expected by chance in the reconstructed assemblage was estimated by randomizing both core and satellite trees 10 thousand times with TreeMap applying the proportional from distinguishable method for generating random trees, and using the machine system clock as a random seed generator.

### The dynamics of gene gain and loss events

In addition to the study of the highly conserved core of DNA viruses and its relationship to the genome as a whole, we also considered evidence that viral protein-coding genes are acquired from host cells. These “satellite” functions are an essential source of evolutionary novelty for RNA viruses [43] and DNA viruses. We consider independent gain and loss events in the Baculoviruses [44], [13], [45], [14], Poxviruses [2] Herpesviruses [1] and Ascoviruses (see below). To investigate the quantitative aspect of convergence of gene acquisition among DNA viruses, we inferred gene gain and loss events with MacClade 4.07 [46], reconstructed along the branches of dendograms calculated from complete genomes. For this purpose we used gene-content tables for DNA viruses built by Herniou *et al.*, [3], [4] that we further extended and modified by Lauzon *et al.*, [45], Oliveira *et al.*, [14] and Wolff *et al.*, [15].
